# Retrograde femoral nailing for deformity correction and fracture treatment in osteogenesis imperfecta: clinical and radiological assessment of a novel technique

**DOI:** 10.1051/sicotj/2025020

**Published:** 2025-04-17

**Authors:** Samuel Georges, Ibrahim Saliba, Georges Finidori, Edouard Haumont, Stephanie Pannier, Zagorka Pejin

**Affiliations:** 1 Orthopedic Department, Necker Hospital 149 Rue de Sèvres 75015 Paris France; 2 Genetics Department, Paris Cité University, INSERM UMR 1163, Imagine Institute 75015 Paris France

**Keywords:** Osteogenesis imperfecta, Retrograde femoral nailing, Femoral deformities, Fractures, Telescoping nails

## Abstract

*Introduction*: Intramedullary anterograde femoral nailing is a standard treatment for femoral deformity and fracture in osteogenesis imperfecta (OI). This study evaluates the clinical and radiological outcomes of a novel retrograde femoral nailing technique. *Methods*: A retrospective analysis was performed on 31 patients with OI who underwent retrograde femoral nailing using Dubow–Bailey nails from 2004 to 2019. A total of 54 femurs were treated for femoral deformity or fracture by three senior surgeons, with a mean follow-up of 2.7 years. Clinical outcomes, including knee range of motion and pain, were assessed. Radiological outcomes included deformity angle (DA), neck shaft angle (NSA), mechanical lateral distal femoral angle (mLDFA), and nail positioning on AP and lateral X-rays. Potential complications, including hip osteonecrosis, distal femoral growth arrest, and infections, were also evaluated. *Results*: The procedure showed favorable outcomes, with no postoperative knee motion limitations or persistent pain. The mean pre-operative DA on AP and lateral views was 29° and 40°, respectively, with no residual deformity after surgery. The mean NSA and mLDFA were 132° and 101° before surgery, compared to 143° and 89° post-operatively. Nail alignment was optimal in 81% of the femurs, with proper positioning in both the distal epiphysis and femoral neck. No cases of hip osteonecrosis, distal femoral growth arrest, or infection were reported. Hardware migration occurred in seven cases. *Conclusion*: Retrograde femoral nailing is a safe and effective technique for managing femoral deformities and fractures in OI.

## Introduction

Osteogenesis Imperfecta (OI) is a genetic disorder caused by mutations in the COL1A1 or COL1A2 genes, leading to alterations in type I collagen [1, 2]. Its incidence ranges from 1 in 10,000 to 1 in 20,000 live births [3]. Patients with OI typically experience early-onset recurrent fractures and progressive bony deformities, often requiring realignment osteotomies and internal fixation [4].

Anterograde femoral nailing is the gold standard for femoral deformity correction in OI patients [4]. Since the original description of the Bailey and Dubow telescopic rod system in 1963 [5], numerous implant designs have been introduced to improve fixation and longevity [6, 7]. However, complications remain common, including gluteal muscle weakness, Trendelenburg gait, insufficient correction of the neck-shaft angle (NSA), eccentric nail positioning, femoral head osteonecrosis, nonunion, nail migration, and infection [8–13]. Many of these complications stem from the anterograde approach itself, which poses challenges in achieving optimal implant positioning while preserving surrounding structures.

To address these limitations, we developed a retrograde femoral nailing technique using the Bailey and Dubow telescopic rod system. Originally described by co-author GF in 1989, this technique has been the standard approach for OI patients in our institution. It was designed to optimize nail positioning along the femur, improve deformity correction, and reduce complications associated with anterograde nailing, particularly those related to hip mechanics, growth plate disruption, and femoral head vascularization.

Despite the long-standing use of this technique in our institution, no studies have systematically evaluated its outcomes compared to anterograde nailing. This study aims to fill this gap by assessing the clinical and radiographic outcomes of retrograde femoral nailing in OI patients. By doing so, we seek to determine whether this approach offers a viable alternative with fewer complications and improved deformity correction.

## Materials and methods

### Study design

This retrospective study was approved by the ethical committee of our institution. We reviewed medical records and radiographs of OI patients who underwent retrograde intramedullary nailing with Bailey and Dubow telescopic rods for femoral fracture or deformity between 2004 and 2019 at a pediatric university hospital. All surgeries were performed by three senior orthopedic surgeons.

### Patient population

A total of 31 patients met the inclusion criteria, which required that they had no prior femur surgeries and underwent retrograde femoral nailing for fracture or deformity. The minimum follow-up period was 48 months or until an adverse event, such as a fracture around the nail requiring exchange, occurred.

Among these patients, 13 (42%) were female, with a mean age at surgery of 3.1 years and a mean follow-up of 2.7 years. The identified genetic mutations were primarily COL1A1 and COL1A2. Table 1 summarizes the demographic and clinical data.

**Table 1:** This table summarizes all the characteristics and demographic data of this study population.

Patient	Side	Sex	Surgery date	Age at date of surgery	Indication of surgery	Classification of silence [14]	Gene	Pre Op: NSA	Pre op: deformity Angle AP	Pre op: Deformity Angle L	Pre op: mLDFA
1	R	M	03/09/04	4	Deformity	4	Col1A1	130	26	30	103
1	L	M	17/02/04	4	Deformity	4	Col1A1	139	16	68	105
2	R	M	23/03/16	1.75	Deformity	3	Col1A2	131	N	35	97
2	L	M	08/09/16	2	Deformity	3	Col1A2	110	32	32	92
3	L	M	05/11/13	3	Deformity	3	Col1A2	117	38	45	113
3	R	M	06/12/13	3	Deformity	3	Col1A2	114	38	N	105
4	L	M	20/02/19	1.9	Fracture/Deformity	1	Col1A2	119	29	10	117
4	R	M	24/07/19	2	Fracture/Deformity	1	Col1A2	117	37	8	125
5	R	M	25/01/20	2	Fracture/Deformity	5	IFITM5	143	55	N	109
5	L	M	13/07/20	2	Deformity	5	IFITM5	144	18	N	101
6	R	F	12/09/13	3	Fracture	5	IFITM5	155	15	16	92
7	R	M	10/05/13	3	Deformity	3	WNT1	150	62	92	118
7	L	M	12/07/13	3	Deformity	3	WNT1	160	71	99	113
8	L	F	25/03/16	3	Fracture	4	Col1A2	140	20	N	95
8	R	F	27/04/16	3	Deformity	4	Col1A2	150	57	18	111
9	R	F	23/07/09	1	Fracture	3	Col1A2	134	32	18	110
9	L	F	17/09/09	1	Deformity	3	Col1A2	117	9	62	100
10	L	F	02/11/15	6	Fracture	4	NI	146	3	36	94
10	R	F	02/05/14	4	Fracture	4	NI	145	15	45	84
11	R	M	08/03/17	2	Deformity	4	Col1A2	140	62	30	113
11	L	M	26/04/17	2	Deformity	4	Col1A2	157	29	5	104
12	L	M	26/04/17	2	Deformity	1	CRTAP	137	22	46	96
13	R	F	26/11/18	1	Deformity	6	SERPFIN1	139	20	35	108
13	L	F	24/12/18	1	Deformity	6	SERPFIN1	149	45	30	120
14	R	F	01/06/15	9	Deformity	5	IFITM5	107	10	16	91
15	L	M	07/01/16	11	Fracture	4	Col1A1	142	0	19	88
15	R	M	27/02/16	11	Deformity	4	Col1A1	140	27	20	108
16	L	F	23/04/17	1	Deformity	3	NI	102	2	37	111
16	R	F	30/05/17	1	Fracture	3	NI	110	5	30	95
17	R	M	31/08/12	1	Deformity	3	Col1A1	116	4	67	96
17	L	M	22/01/13	2	Deformity	3	Col1A1	112	10	83	90
18	R	M	18/07/16	4	Deformity	3	FKBP10	122	20	32	105
18	L	M	28/09/16	4	Deformity	3	FKB510	109	7	40	101
19	L	F	20/04/12	2	Fracture	3	Col1A2	131	134	136	101
19	R	F	13/07/12	3	Deformity	3	Col1A2	118	134	122	99
20	L	M	05/06/19	4	Deformity	3	Col1A1	111	14	65	102
20	R	M	01/07/19	4	Deformity	3	Col1A1	130	54	70	116
21	L	F	01/06/16	3	Fracture	1	Col1A2	130	0	8	86
22	L	M	13/10/08	8	Fracture	4	NI	132	10	8	83
23	L	F	05/04/12	3	Deformity	3	MESDC2	131	57	59	93
23	R	F	05/04/12	3	Deformity	3	MESDC2	137	56	52	89
24	L	F	04/06/18	4	Fracture	1	Col1A1	142	0	0	86
25	L	F	29/01/16	2	Deformity	6	SERPFIN1	137	33	73	102
25	R	F	14/12/15	2	Deformity	6	SERPFIN1	145	50	88	118
26	L	F	03/12/18	3	Fracture	3	Bruck	130	10	8	84
26	R	F	24/12/18	3	Deformity	3	Bruck	133	8	10	86
27	R	M	15/05/19	3	Fracture	1	Col1A1	140	0	8	88
28	L	M	20/08/10	1.5	Deformity	3	Col1A1	114	N	22	92
28	R	M	04/06/10	1.5	Deformity	3	Col1A1	120	56	36	97
29	R	M	16/03/06	1.9	Fracture	3	Col1A1	128	15	40	105
29	L	M	17/11/06	2	Fracture	3	Col1A1	152	30	42	109
30	R	M	04/04/17	5	Fracture	4	Col1A1	140	0	0	87
31	R	M	18/02/13	2	Deformity	3	Col1A1	145	4	34	98
31	L	M	17/05/13	2	Fracture	3	Col1A1	154	5	30	128

NI = Not identified.

### Hardware description

**Figure 1:**
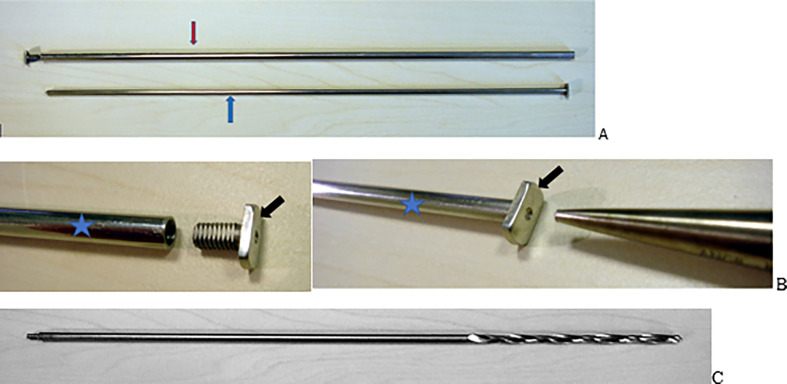
Description of the telescopic nail utilized in this study. A: Red arrow: The female rod; Blue arrow: The male rod. B: The T-piece (black arrow) is secured by screwing it into the female rod (Blue star). C: The drill bit.

The implant used was a modified Bailey and Dubow telescopic nail (Medicalex). The nail consists of a female component (Figure 1A) with threading at one end to ensure secure attachment to the drill bit, facilitating insertion into the femur. A T-piece (Figure 1B) is secured by screwing it into the female component, and forceps are used to tightly clamp it, preventing loosening. Additionally, the male component (Figure 1A) also ends in a T-shaped piece. The nail is available in diameters of 3.5, 4, 4.5, 5, and 6 mm.

The primary instrument for this retrograde technique is a long drill bit (Figure 1C). One end is reamed 0.2 mm larger than the nail diameter, allowing for preparation of the medullary canal and precise, perpendicular osteotomies aligned with the knee joint. The opposite end of the bit is threaded to secure the female part of the nail, guiding its insertion. Additional impactors for the T-pieces complete the instrument set. Custom nail planning is based on an AP or lateral X-ray of the femur, using a radiopaque index for accurate magnification assessment.

### Surgical technique

The procedure was performed under general anesthesia with the patient in the lateral decubitus position. The affected limb was prepared and draped up to the hip. The image intensifier was positioned horizontally to allow full visualization of the femur.

**Figure 2:**
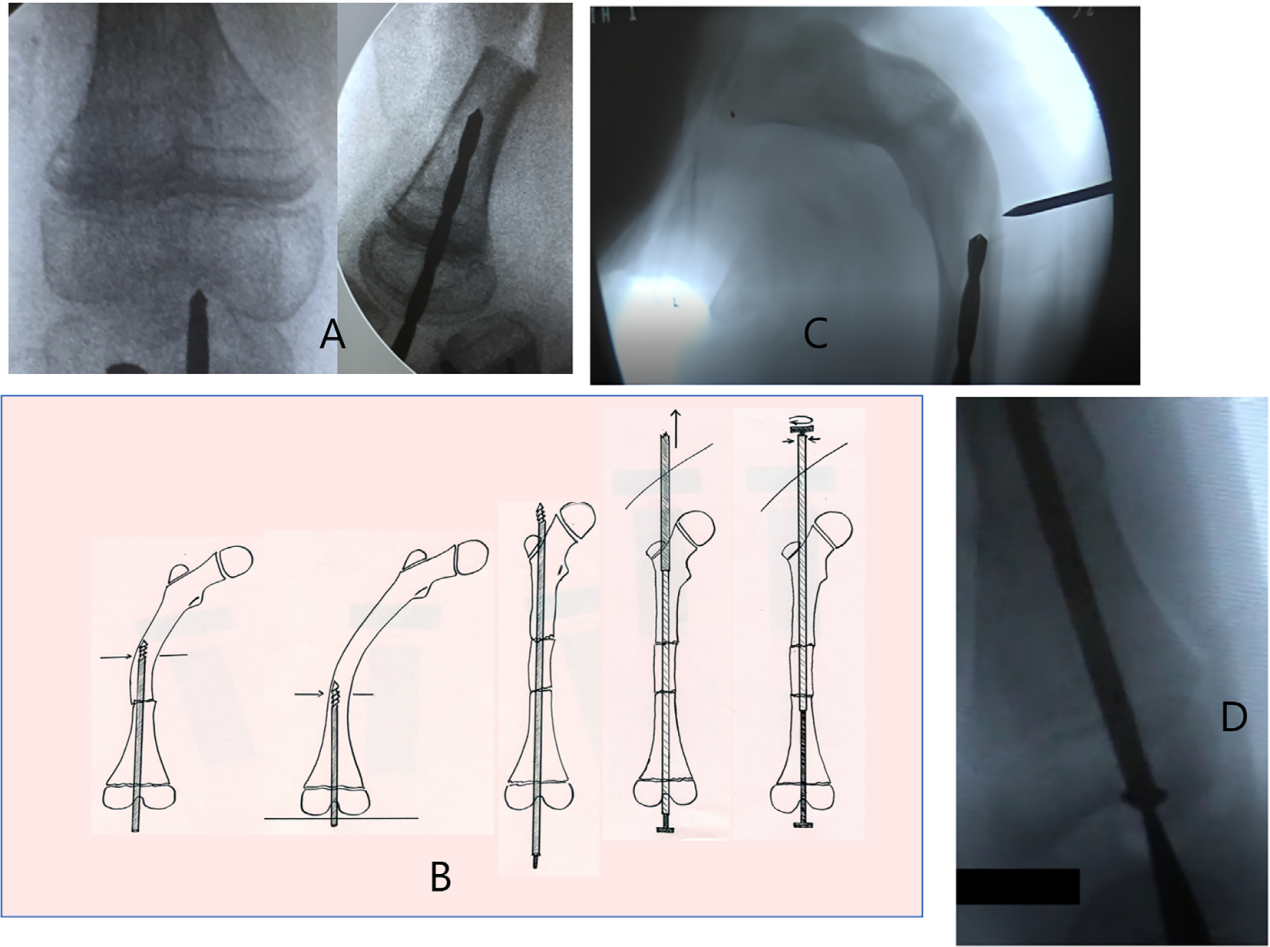
This figure illustrates the surgical technique. A: Manual drilling is performed perpendicular to the joint line in both anterior (left) and lateral (right) views. B: During drilling, it is crucial to remain perpendicular to the distal femoral joint line, performing osteotomies as needed. These can be done either via percutaneous osteoclasis or through a lateral open approach (this figure was created by the authors to be used in this article). C: The exit point for the drill bit is at the femoral neck, allowing for valgus fixation to prevent coxa vara deformity and the risk of lateral nail exit or femoral neck fracture. To facilitate this, a subtrochanteric osteotomy may be performed. D: The male component is introduced percutaneously through the knee and impacted into the distal femoral epiphysis.

The drill bit is introduced at the knee through a percutaneous transpatellar tendon approach, using a T-handle. After confirming correct positioning on both anterior and lateral views with the image intensifier, the drill bit is advanced into the center of the intercondylar notch. This manual drilling is performed perpendicular to the joint line in both anterior and lateral views (Figure 2A). During drilling, it is crucial to remain perpendicular to the distal femoral joint line, performing osteotomies as needed (Figure 2B). These can be done either via percutaneous osteoclasis or through a lateral open approach. For surgical exposure, dissection and periosteal stripping are primarily performed with electrocautery to limit bleeding, especially in severe cases. The osteotomy is performed with an oscillating saw to prevent splintering of the bone and to ensure a precise osteotomy.

The exit point for the drill bit is at the femoral neck, allowing for valgus fixation to prevent coxa vara deformity and the risk of lateral nail exit or femoral neck fracture. To facilitate this, a sub trochanteric osteotomy may be performed (Figure 2C). The drill bit is then retracted percutaneously at the level of the gluteal region. The female component of the telescopic nail, with the male component inside, is screwed onto the drill, guiding it to the proximal exit point. The T-tool is screwed and secured in the female component to prevent unscrewing, and the entire assembly is impacted at the superior border of the femoral neck. The male component is introduced percutaneously through the knee and impacted into the distal femoral epiphysis (Figure 2D).

Postoperatively, the limb was immobilized with a hip spica cast for 4 weeks in cases of percutaneous osteotomy and 6 weeks for open osteotomy, particularly in valgus correction cases. This was followed by intensive physiotherapy focusing on hip and knee range of motion. Radiographs were obtained at 6 weeks, 12 weeks, 24 weeks, 48 weeks, and annually thereafter.

### Outcome measures

#### Clinical outcomes

Knee range of motion (ROM) was assessed preoperatively and at the last follow-up. A ROM between 0° and 130° was considered normal [15].

Pain levels were evaluated using the Visual Analog Scale (VAS) [16].

#### Radiological outcomes

Radiographic assessments were performed preoperatively, after cast removal (4–6 weeks), and at the last follow-up.

**Figure 3:**
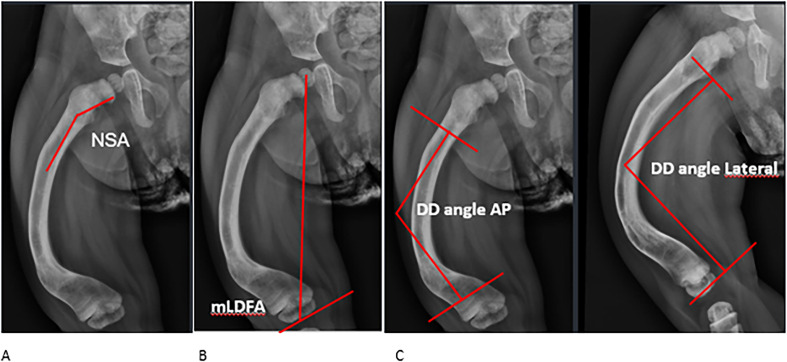
This figure illustrates the measured angles which are utilized as radiological outcomes. A: The neck-shaft angle (NSA) measured on AP radiographs. Normal range: 120–140 degrees. B: mLDFA was defined as the angle measured between the mechanical axis of the femur and the knee joint line. Normal range: 85–90 degrees. C: The femoral deformity angle (DA) was defined as the angle measured between two lines drawn along the bone shaft axis and intersecting at the apex of the angulation on AP (left) and Lateral (right) view.

The neck-shaft angle (NSA) was measured on AP radiographs, with values between 120° and 140° considered normal. Coxa vara was defined as an NSA < 120°, while coxa valga was defined as an NSA > 140° (Figure 3A) [17].

The mechanical lateral distal femoral angle (mLDFA) was assessed, with normal values ranging between 85° and 90° (Figure 3B) [18]. The deformity angle (DA) was determined by measuring the intersection of two shaft axis lines on both AP and lateral views (Figure 3C) [19].

**Figure 4:**
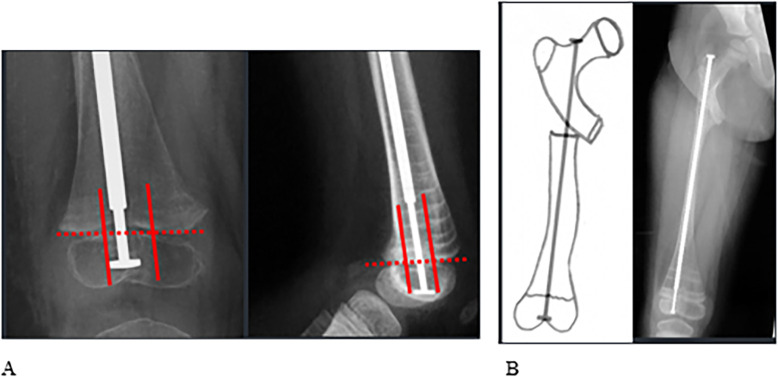
This figure shows optimal positioning of the hardware in the proximal and distal femur. A: In order to define the optimal position of the nail distally, we virtually divided the distal femoral epiphysis into three compartments. A good positioning was defined as the distal end of the male rod passing through the middle compartment of the epiphysis on AP (left) and lateral (right) X-rays. B: Proximally, the position was considered optimal when the female rod exited through the femoral neck.

The positioning of the nail was examined at the hip and knee joints on AP and lateral radiographs. The distal positioning was deemed optimal when the male rod passed through the middle compartment of the epiphysis (Figure 4A), while the proximal positioning was ideal when the female rod exited through the femoral neck (Figure 4B). Any deviation from these ideal positions was categorized as eccentric. The number of osteotomies required for deformity correction was also recorded based on postoperative X-ray analysis.

#### Statistical analysis

Statistical analyses were conducted using R software (version 3.5.0), accessed on September 5, 2024, at https://www.R-project.org. Quantitative variables were expressed as means and standard deviations, while categorical variables were presented as frequencies and percentages. Comparisons between two groups were performed using the Mann–Whitney test, and comparisons among more than two groups were carried out using the Kruskal–Wallis test. A *p*-value of less than 0.05 was considered statistically significant.

## Results

### Clinical outcomes

Postoperatively, knee range of motion (ROM) was preserved in all patients. Among the 34 femurs without fractures at the time of surgery, preoperative ROM was within the normal range of 0 to 130 degrees. However, ROM assessment was not possible for the 20 femurs with fractures preoperatively, so these were excluded from analysis.

Postoperative pain, assessed using the Visual Analog Scale (VAS), showed a significant reduction over time. The mean VAS score was 6 in the early postoperative period and decreased to 0 at the final follow-up, indicating complete pain resolution. These clinical outcomes are summarized in Table 2.

**Table 2:** This table details the clinical and radiological outcomes of this study population.

Patient	Side	VAS pre-op	VAS post-op	Post op: knee ROM	Total number of osteotomies	Percuta-neous osteocla-sis	Open osteoto-my	POST OP: NSA	POST OP: mLDFA	Proxi-mal exit site (N/GT)	POST OP: coronal positioning in distal epiphysis (Lateral 1/3, Middle 1/3, Medial 1/3)	POST OP: sagittal positioning in distal epiphysis (Anterior 1/3, Middle 1/3, Posterior 1/3)
1	R	0	0	Normal	1	0	1	140	85	N	Middle 1/3	Middle 1/3
1	L	2	0	Normal	2	0	2	142	88	N	Middle 1/3	Middle 1/3
2	R	0	0	Normal	3	2	1	150	91	N	Middle 1/3	Middle 1/3
2	L	3	0	Normal	2	2	0	142	85	N	Middle 1/3	Middle 1/3
3	L	1	0	Normal	2	0	2	130	90	N	Middle 1/3	Middle 1/3
3	R	2	0	Normal	1	0	1	150	91	N	Middle 1/3	Middle 1/3
4	L	8	0	Normal	1	1	0	150	90	N	Middle 1/3	Middle 1/3
4	R	7	0	Normal	2	2	0	130	89	N	Middle 1/3	Middle 1/3
5	R	9	0	Normal	2	1	1	163	90	N	Middle 1/3	Middle 1/3
5	L	0	0	Normal	2	0	2	147	89	N	Middle 1/3	Middle 1/3
6	R	9	0	Normal	0	0	0	155	86	N	Middle 1/3	Middle 1/3
7	R	1	0	Normal	3	3	0	148	89	N	Middle 1/3	Anterior 1/3
7	L	1	0	Normal	2	1	1	147	89	N	Middle 1/3	Middle 1/3
8	L	8	0	Normal	1	1	0	145	88	N	Middle 1/3	Middle 1/3
8	R	0	0	Normal	2	0	2	157	88	N	Middle 1/3	Middle 1/3
9	R	7	0	Normal	1	1	0	143	89	N	Middle 1/3	Middle 1/3
9	L	2	0	Normal	2	2	0	145	95	N	Middle 1/3	Middle 1/3
10	L	9	0	Normal	1	0	1	144	88	N	Middle 1/3	Middle 1/3
10	R	10	0	Normal	1	0	1	149	84	N	Middle 1/3	Middle 1/3
11	R	1	0	Normal	2	1	1	156	89	N	Middle 1/3	Middle 1/3
11	L	0	0	Normal	1	0	1	157	89	N	Middle 1/3	Middle 1/3
12	L	0	0	Normal	1	0	1	151	90	N	Middle 1/3	Middle 1/3
13	R	2	0	Normal	3	2	1	139	92	N	Middle 1/3	Middle 1/3
13	L	1	0	Normal	2	2	0	148	102	N	Middle 1/3	Middle 1/3
14	R	1	0	Normal	2	2	0	137	89	N	Lateral 1/3	Middle 1/3
15	L	10	0	Normal	1	0	1	148	84	N	Middle 1/3	Middle 1/3
15	R	0	0	Normal	3	1	2	152	88	N	Middle 1/3	Middle 1/3
16	L	0	0	Normal	2	1	1	130	91	N	Middle 1/3	Middle 1/3
16	R	9	0	Normal	2	1	1	127	92	N	Middle 1/3	Middle 1/3
17	R	0	0	Normal	2	1	1	130	89	N	Middle 1/3	Middle 1/3
17	L	1	0	Normal	2	1	1	125	95	N	Middle 1/3	Middle 1/3
18	R	1	0	Normal	1	0	1	142	90	N	Middle 1/3	Middle 1/3
18	L	1	0	Normal	1	0	1	123	87	GT	Middle 1/3	Middle 1/3
19	L	8	0	Normal	1	0	1	145	89	N	Middle 1/3	Middle 1/3
19	R	0	0	Normal	1	0	1	104	89	N	Middle 1/3	Middle 1/3
20	L	2	0	Normal	1	0	1	130	90	N	Middle 1/3	Middle 1/3
20	R	0	0	Normal	1	0	1	129	94	N	Lateral 1/3	Middle 1/3
21	L	10	0	Normal	1	0	0	148	87	N	Middle 1/3	Middle 1/3
22	L	10	0	Normal	1	0	1	145	86	N	Middle 1/3	Anterior 1/3
23	L	1	0	Normal	2	1	1	148	90	N	Lateral 1/3	Middle 1/3
23	R	1	0	Normal	2	1	1	155	89	N	Lateral 1/3	Middle 1/3
24	L	8	0	Normal	1	0	1	150	87	N	Middle 1/3	Middle 1/3
25	L	3	0	Normal	3	2	1	130	91	N	Lateral 1/3	Anterior 1/3
25	R	2	0	Normal	3	2	1	145	90	N	Lateral 1/3	Anterior 1/3
26	L	9	0	Normal	1	1	0	143	87	N	Middle 1/3	Middle 1/3
26	R	1	0	Normal	0	0	0	129	90	GT	Middle 1/3	Middle 1/3
27	R	9	0	Normal	1	0	0	140	89	N	Middle 1/3	Middle 1/3
28	L	0	0	Normal	2	1	1	148	87	N	Middle 1/3	Middle 1/3
28	R	0	0	Normal	2	0	2	127	90	N	Middle 1/3	Middle 1/3
29	R	10	0	Normal	1	0	1	147	92	N	Middle 1/3	Middle 1/3
29	L	10	0	Normal	2	0	2	150	89	N	Middle 1/3	Middle 1/3
30	R	8	0	Normal	1	0	1	147	85	N	Middle 1/3	Middle 1/3
31	R	0	0	Normal	2	1	1	158	90	N	Middle 1/3	Middle 1/3
31	L	9	0	Normal	3	1	2	157	90	N	Middle 1/3	Middle 1/3

### Radiological outcomes

The correction of femoral deformity was reflected in significant improvements in radiographic parameters. The NSA increased from a preoperative mean of 132 degrees to 142 degrees immediately postoperatively. At the final follow-up, the NSA was measured at a mean of 138 degrees. The difference between preoperative NSA and early postoperative NSA was statistically significant (*p* = 0.0001), as was the difference between preoperative NSA and final follow-up NSA (*p* = 0.018). However, the difference between early postoperative and final follow-up NSA was not statistically significant (*p* = 0.07).

Similarly, the mLDFA showed a significant correction, decreasing from a preoperative mean of 101 degrees to 89 degrees postoperatively. At the final follow-up, the mean mLDFA was 91 degrees. The difference between preoperative and early postoperative mLDFA was highly significant (*p* = 8.4e–9), as was the difference between preoperative and final follow-up mLDFA (*p* = 4.3e–7). However, no significant change was observed between the early postoperative and final follow-up mLDFA values (*p* = 0.49).

Femoral deformity was also evaluated using the deformity angle (DA), which was 29 degrees on AP X-rays and 40 degrees on lateral X-rays preoperatively. Postoperatively, no residual deformity was observed in any patient on both AP and lateral X-rays through the final follow-up.

Implant positioning was satisfactory in the majority of cases. On postoperative AP X-rays, the nail position was deemed satisfactory in 48 out of 54 femurs (89%), and on lateral X-rays, it was satisfactory in 50 out of 54 femurs (93%). The mean number of osteotomies required to achieve proper alignment of the femoral deformity was 1.6 per femur. A summary of these radiological outcomes is presented in Table 2.

### Complications

Complications were observed in a subset of patients. The most common complication was an inability of the nail to telescope, occurring in 28 out of 54 femurs (52%). Proximal hardware migration was reported in 7 out of 54 femurs (13%). Importantly, no cases of intraarticular migration into the knee joint were observed. Additionally, there were no recorded instances of growth arrest, aseptic necrosis, nonunion, or infection.

## Discussion

Osteogenesis imperfecta (OI) is a rare genetic disorder characterized by bone fragility, recurrent fractures, and progressive deformities [1, 2]. The primary goal of surgical management in these patients is to correct deformities, stabilize fractures, and improve functional mobility while minimizing complications. The main finding of this study is that retrograde nailing of the femur in patients with OI achieved effective deformity correction and proper fracture alignment. The procedure ensured optimal positioning and secure anchoring of the nail at both ends of the femur. Notably, there was no risk of growth arrest in growing children.

The primary limitation of this study is its retrospective design and the absence of a control group, which limits the ability to establish causality and compare outcomes with a standardized baseline. However, despite this limitation, our study population is notably heterogeneous, encompassing a diverse range of OI patients across different age groups, varying levels of walking and functional abilities, and multiple OI phenotypes. This diversity adds to the generalizability of the findings but also introduces variability that could affect the outcomes. Another limitation of this study is that we did not analyze the results following the replacement of the first nail. Additionally, our focus was not on disease-specific factors inherent to osteogenesis imperfecta (OI), but rather on the surgical technique itself.

The clinical outcomes were satisfactory in our study population with an improvement in motor function and the achievement of a normal ROM following surgery. Conversely, previous studies [20, 21] reported that retrograde femoral nailing has been demonstrated to be a safe alternative to anterograde nailing, although it is known to cause knee pain, which is reported as one of the most common adverse effects of retrograde nailing, with incidence rates as high as 70%. In our series, no knee pain was reported during long-term follow-up. We believe this is likely due to the small diameter of the telescopic nail, femoral notch enter point and the high potential for fibrocartilage tissue formation in children. Moreover, the drill bit pass through only once, with no reaming required, eliminating the risk of reaming debris entering the knee joint, as the nail is directly connected to the drill bit. Knee pain was not evaluated at the 6-week follow-up, as spica cast removal is often associated with knee and hip apprehensions in children. No knee pain was reported at the last follow-up.

We hypothesize that restoring the proper mechanics of the hip and knee joints will reduce the risk of pathological fractures and enhance functional outcomes.

Regarding the hip joint, we realized corrective osteotomies of the coxa vara with a target NSA of more than 135 degrees in order to prevent deformity recurrence over time. To achieve this goal, the nail was fixed proximally through femoral neck. We measured the NSA as an indicator of optimal correction and as an indicator of the static and mechanical properties of the joint. Our findings indicate that this procedure results in normal to relatively increased NSA, which is crucial in preventing postoperative complications and unfavorable outcomes. As per Aarabi et al. [12], the clinical consequences of coxa vara are well established; a reduction in the neck–shaft angle shortens the abductor lever arm, resulting in abductor insufficiency and a positive Trendelenburg sign. Notably, telescopic nails are straight; therefore, in cases of antegrade nailing with a greater trochanteric entry point, aiming for the center of the distal femoral physis can result in fixation of the femoral neck in varus, increasing the risk of worsening the deformity. Conversely, if the femoral neck is positioned in valgus, the distal nail may end up in the medial femoral condyle, raising the risk of epiphysiodesis.

Furthermore, the retrograde telescopic nailing procedure does not compromise the gluteal muscles, which are vital for optimal hip function.

In our study, no cases of femoral head osteonecrosis were observed, likely due to several key aspects of our surgical technique. By passing through the femoral neck without reaming and advancing the drill bit solely through the neck, we minimized mechanical and thermal stress on the surrounding bone and vascular structures. This approach helped preserve the femoral head’s blood supply by preventing thermal effects and avoiding ischemia that could result from motorized drilling. Additionally, the use of a direct connection between the nail and the drill bit further reduced the risk of vascular disruption and thermal damage. These procedural factors likely contributed to the preservation of femoral head vascularity, potentially explaining the absence of osteonecrosis in our patient cohort.

The most widely recognized surgical method for treating femoral fractures or deformities in patients with OI is based on the antegrade femoral nailing technique [4–6]. However, this method is associated with several complications, including Trendelenburg gait, persistent hip pain, proximal rod migration, difficulties in correcting distal femoral deformities, and eccentric positioning of the distal end in the knee due to the trochanteric entry point, which can result in growth disturbances, rod bending, and fractures [8, 9, 13, 22, 23]. A crucial finding of this study was the successful achievement of centered nail positioning on both AP and lateral X-rays in the vast majority of patients.

To the best of our knowledge, this is the first study to describe the retrograde nailing technique using a telescopic rod in patients with OI. Previous research has demonstrated improved motor skills and walking abilities, along with satisfactory correction of deformities, using anterograde nailing [4–7, 22]. However, these studies have reported various complication rates and have questioned the efficacy of these surgical interventions in enhancing functional status and reducing the risk of fractures [8, 9, 24]. In our study, postoperative complications such as intraarticular migration, growth arrest, and aseptic necrosis of the femoral head were absent, and the only reported complications were related to the hardware utilized in this study population.

Furthermore, retrograde nailing demonstrates greater efficiency and ease in correcting distal deformities, although it may lead to distal femoral growth arrest. Our findings suggest that the retrograde technique achieves satisfactory correction. The correction was easier from distal to proximal, allowing for precise alignment. Moreover, the drill bit serves both as a correction tool and as a guide for nail insertion.

**Figure 5:**
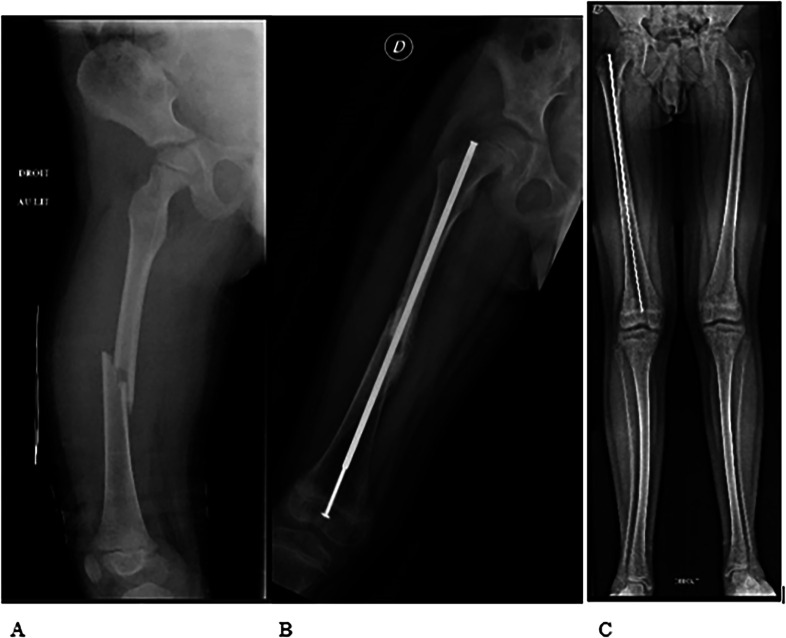
Pre-operative (A), post-operative (B) and 6-year-follow-up (C). X-rays of one of this study population patients showing no growth arrest or aseptic femoral head necrosis along as good nail positioning and femur alignment.

It is crucial to highlight the significance of an accurate entry portal, which ensures optimal implant placement, thereby facilitating effective correction of distal deformities and enabling subsequent proper osteotomies and alignment. Proper alignment of the nail within the center of the knee and femoral neck is essential for stability, achieving satisfactory correction, and preventing growth plate arrest or disturbances. In our study, no growth disturbances were observed and good correction was achieved (Figure 5).

Previous studies [9, 19] have reported that revisions were necessary in 13–53% of patients who received anterograde telescopic nails for femoral fractures, due to rod bending and rod migration. In our series, we achieved excellent anchoring with only 13% migration. However, we observed 52% inability to telescope of the nail. Additionally, we observed no rod bending or non-traumatic femoral fractures at the last follow-up.

The lack of rotational control of the femur and the thin diameter of OI long bones make intramedullary rodding insufficient for providing optimal fixation. Therefore, spica casting is crucial to counteract this mechanical disadvantage and control rotational forces. It also prevents malrotation of bony unions while reducing the postoperative pain. It is important to note that mechanics alone will not prevent fractures, as OI is a congenital disorder caused by mutations in the COL1A1 or COL1A2 genes [1, 2], resulting in abnormal collagen cross-linking and a decrease in type I collagen. This collagen disorder increases the risk of fragility fractures and bone deformities. Therefore, a multidisciplinary approach, including medical treatment with bisphosphonates, is necessary to help reduce fracture risk [25].

In conclusion, the present study demonstrated that retrograde femoral nailing utilizing the Bailey and Dubow telescopic rod system is a safe and effective method for managing fractures and deformities in patients with OI. Satisfactory clinical and radiological outcomes were achieved while no major complications were reported. Modifying and adapting the Bailey and Dubow nail to minimize hardware-related complications has the potential to improve clinical outcomes in the OI population.

## Data Availability

No data allowing patient identification is published.
